# Global brain banking challenges through the lens of Southeast Asia’s inaugural brain bank

**DOI:** 10.3389/fneur.2026.1768688

**Published:** 2026-02-13

**Authors:** Priscilla Martin, Moses Tandiono, Apphia Choa, Jia Nee Foo, Tianrong Yeo, Richard Reynolds, Adeline Su Lyn Ng

**Affiliations:** 1 Brain Bank Singapore, Singapore, Singapore; 2 Lee Kong Chian School of Medicine, Nanyang Technological University, Singapore, Singapore; 3 Department of Neurology, National Neuroscience Institute, Singapore, Singapore; 4 Duke-NUS Medical School, Singapore, Singapore; 5 Division of Neuroscience, Department of Brain Sciences, Imperial College London, Hammersmith Hospital Campus, London, United Kingdom

**Keywords:** biobanking and biorepositories, brain banking, brain donor, neurodegenerative disease, Southeast Asia

## Abstract

Brain Bank Singapore (BBS) is the first and only national brain donation programme in Southeast Asia. This perspective article explores the challenges that have risen from establishing an ethically governed post-mortem brain bank in a small multicultural society like Singapore. These experiences reflect the complex realities of developing brain banks in culturally diverse settings and the strategies essential for creating sustainable and internationally aligned repositories.

## Introduction

International brain banks continue to play a critical role in neuroscience research by facilitating joint research efforts globally through the distribution of high-quality post-mortem brain tissues. These consortia have evolved strategically, enabling individual banks to focus on specific brain diseases. Like many such repositories worldwide, they face ongoing constraints involving donor numbers, safety requirements, and the logistical intricacies of maintaining a functioning brain bank (1). Despite such advancements, there seems to be an uneven distribution of resources across the globe with the majority of brain banks concentrated in North America (86) and Europe (35), and only 6 in Asia (2) and none in Southeast Asia. Hence, the establishment of a brain bank in Singapore in 2019 addressed this shortfall, pioneering the first and only initiative of its kind within the region and enabling the collection of tissues crucial for developing targeted therapies and medical advancements for Southeast Asian populations in the field of neuroscience.

## Background

BBS was established through a joint partnership between Lee Kong Chian (LKC) School of Medicine (medical school of the Nanyang Technological University), the National Neuroscience Institute (Singapore’s national specialist centre for conditions of the brain, spine, nerve and muscle) and the National Healthcare Group (a leading public healthcare provider), to create a national tissue repository for post-mortem brain tissues and spinal cords. The National University of Singapore’s Yong Loo Lin School of Medicine joined as a partner in 2021, followed by Duke-NUS Medical School and the Institute of Molecular and Cell Biology (an autonomous research institute of the Agency for Science, Technology and Research) in 2024 as the 5th and 6th partners, respectively. BBS operates a nationwide pre-consented brain donation programme, collecting and preserving brain tissues from individuals with neurological and psychiatric disorders as well as healthy individuals.

## Internationalisation and collaboration

From its inception, BBS was founded through the joint leadership of both local and international experts. The founding Director of the Multiple Sclerosis and Parkinson’s Tissue Bank at Imperial College London, Professor Richard Reynolds, also served as the founding director of BBS. Drawing on Imperial’s long-standing expertise in brain tissue banking, BBS adapted its standard operating procedures to align with internationally recognised practices for tissue collection, processing, and storage. This collaboration has facilitated both the transfer of technical knowledge and biobanking expertise through BBS staff training and sharing of established protocols, to allow BBS to initiate its activities and establish itself as a national brain bank in Singapore and the Southeast Asian region.

## Donor recruitment challenges

### Religious sensitivities

In Singapore, 75.9% of its population are of Chinese ethnicity (3) amongst whom Buddhism is the most widely practised religion. Traditional Buddhist beliefs maintain that the body should remain intact before the consciousness has fully departed so as not to affect rebirth (4).

However, Buddhist scholars have previously suggested their support for organ donation, viewing it as a generous act that will benefit others after one’s death (5). In Singapore, some Buddhist families observe an 8 h ‘no-touch” period following death during which the body is left undisturbed allowing the consciousness to depart peacefully. There have been cases where BBS have had to honour this custom by delaying retrieval until the completion of this ritual. As this falls well within the recommended 24-h post-mortem interval (PMI) for brain donation, it allows both cultural sensitivity and scientific integrity to be upheld.

Malays are Singapore’s second largest ethnic group, comprising 13.5% of the population, with Islam being the most widely practised religion amongst them (6). Religious sensitivities shape the attitudes toward organ donation amongst the Singaporean Muslims. According to a survey “Perceptions of Organ Donation Among Muslims in Singapore”, 36.7% of respondents were unsure about the Islamic permissibility of organ donation, while 83.7% called for increased public awareness and education (7).

### Cultural barriers

Although the donor’s consent is legally sufficient, family objection at the time of death can make it challenging to proceed in a culturally sensitive manner. When the family perceives that the process of tissue retrieval interferes with funeral arrangements or constrains the time needed for customary rites, they may choose not to proceed with brain retrieval. In such cases, the BBS respects the family’s wishes, resulting in the withdrawal of the intended donation. This is characteristic of a collectivistic society—whereby decisions are largely driven by the family or community’s needs which may be prioritized over individual autonomy (8).

To mitigate this, BBS encourages donors to clearly communicate their end-of-life decisions to their next-of-kin (NOK) to ensure their wishes are respected and fulfilled. Donors and their families are provided with informational resources that provide clarity on the entire donation process, and a meeting or call is arranged to address any concerns prior to obtaining consent. There is also regular engagement with the donor and their family (through bi-annual surveys, annual newsletters, regular social media posts, annual donor engagement events etc) throughout the journey from registering as a donor, to demise.

At the time of death, the donor coordinator will maintain close communication with the NOK to provide support and updates throughout the retrieval process. Every effort is made to accommodate relevant cultural or religious rituals while ensuring PMI is not compromised.

## Legal and procedural barriers

Since 2020, BBS has operated under the Human Biomedical Research Act (HBRA), through which individuals may opt in to donate their brains and cerebrospinal fluid for research. In Singapore, there are two other organ donation frameworks. The Human Organ Transplant Act (9) is an opt-out law that permits the removal of specific organs (kidneys, heart, liver and corners) from citizens and permanent residents aged 21 and above with mental capacity, unless they have formally opted out. The Medical (Therapy, Education and Research) Act is an opt-in scheme that allows individuals to pledge the donation of their organs, tissue or whole body for transplantation, education or research, beyond those covered under HOTA (10). A common misconception amongst members of the public is that pledging whole-body donation under MTERA automatically includes the brain. In practice, the brain is not retrieved for research under MTERA unless explicit consent is provided by registering officially as a brain donor with the BBS.

### Outreach and public education

The BBS recognises its significant role and responsibility in educating the public about neuroscience, neurological and psychiatric disorders. Our regular public outreach efforts have expanded to include collaborations with major hospitals and healthcare institutions, academic institutions like LKC School of Medicine and NUS, and major community partners like the Parkinson Society Singapore, Dementia Singapore and Multiple Sclerosis Society Singapore. These partnerships have given us regular platforms for promoting awareness on neuroscience research and dispel misconceptions on Singapore’s organ donation programmes, through public talks and setting up informational booths at academic symposiums and community events. On average, BBS conducts nearly 30 public talks and outreach activities each year. During these sessions, members of public often ask practical questions about the steps families should take when a registered donor passes on and why the BBS is not automatically notified of a donor’s death through national systems. These engagements not only enhance understanding of brain donation but also provide BBS staff with valuable insight into common concerns and apprehensions surrounding post-mortem organ donation.

Nevertheless, some organisations consider topics related to death and organ donation too sensitive and perhaps even confronting for their audience, often largely comprising of seniors (aged >65 years). To navigate this, we often invite non-profit organisations focused on neurodegenerative disease to speak alongside us. For example, a talk on the prevalence of dementia in Singapore sets the stage to introduce how post-mortem brain analysis can help identify drug targets for reversing dementia, thereby emphasizing the importance of brain donation.

As a relatively new initiative launched shortly before the COVID-19 pandemic, donor recruitment and outreach was significantly restricted during the BBS’ first 2 years of operation. With a median donor age of 58 years, most pledges have not yet been realised, resulting in limited disease-specific material currently available for research. The repository, however, has already been opened to supply tissues for pilot research studies, with the Tissue Access Committee rigorously assessing requests for scientific merit. Despite constrained initial resources, BBS has begun supporting pilot studies with partner institutions which will lay the groundwork for larger disease-focused collaborations in the future.

## Governance and multi-institutional complexity

The governance framework of BBS is one that is multi-institutional, comprising representatives from all six partner institutions structured through the Steering Committee, Executive Committee, Tissue Access Committee and Operations Committee. The Steering Committee provides high-level strategic directions on policy, sustainability planning and plays a pivotal role in partnership expansion. The Executive Committee plays a key role in facilitating collaboration within and across partners institutions while the Operations Committee manages the day-to-day operations including but not limited to donor recruitment, tissue retrieval logistics, outreach activities, and adherence to standardised protocols, ensuring alignment with strategic objectives set by higher-level committees.

## Challenges of infectious disease exclusions: hepatitis B

In line with global tissue banking guidelines, donors with known infectious diseases such as HIV, tuberculosis (TB), Hepatitis B, and prion diseases are routinely excluded from donation to ensure biosafety. One of the most persistent challenges for BBS has been determining how to manage potential donors who are hepatitis B carriers. Chronic hepatitis B affects nearly 300 million people worldwide, with 75% of cases occurring in the Asia-Pacific region; Singapore’s carrier prevalence is about 6% (11) and the prevalence is much higher in older adults, which can be attributed to the fact that Singapore’s hepatitis B vaccination programme was only extended to all newborns under the National Childhood Immunisation Programme in 1987 (12). This prevalence means that strict exclusion of Hepatitis B carriers substantially reduces the donor pool and risks skewing the representativeness of the brain bank’s collection. Furthermore, a number of potential donors did not proceed with officially registering as donors as they already had known Hepatitis B carrier status.

Formalin fixation of whole brains may reduce infectivity but does not guarantee complete inactivation and it is unclear whether viral DNA can remain detectable in the brain (13). Consequently, most established brain banks maintain a blanket exclusion of Hepatitis B carriers. For BBS, this has resulted in the rejection of multiple potential donors, with missed retrievals due to Hepatitis B carrier status (*n* = 3) and COVID-19 infection (*n* = 1), as well as additional missed retrievals attributed to family refusal owing to funeral timing constraints (*n* = 1), lack of manpower at the retrieval site (*n* = 1), coroner’s case (*n* = 1), death overseas (*n* = 1), and a lapse in agreement with the retrieval laboratory (*n* = 1), underscoring the tension between safety, scientific need, and cultural context in regions. This reflects a broader challenge for brain banks in Asia and other regions with higher infection prevalence, where Hepatitis B remains highly prevalent, accounting for roughly 59% of the worldwide burden of chronic diseases in the Asia-Pacific area (14). These uncompromising biosafety standards, though essential, inevitably constrain inclusivity in regions where specific infectious diseases are prevalent.

## Data management and digital transition

One of the key operational challenges faced by BBS was the management and traceability of donor and tissue records. In its initial years, all documentation including donor consent forms, medical questionnaires, and tissue dissection images were stored as soft copies on a shared drive accessible only to authorised brain bank staff, while physical copies were kept in a locked cabinet inside a restricted-access room. While suitable for the BBS’s formative years, this approach proved increasingly inefficient as the donor registry grew. Cross-referencing information between donors, consent forms, and corresponding tissue samples required manual searches, making it difficult to filter and retrieve data efficiently. The lack of an integrated system also limited BBS’s ability to track biospecimen usage, check-in/check-out history, and long-term storage records,

To address these limitations and improve data integrity, BBS implemented the LabVantage Laboratory Information Management System (LIMS) in 2024. The system provides a unified, secure platform that consolidates donor registration details, consent documentation, clinical data, and tissue inventory within a single database. Each tissue sample can now be tracked throughout its lifecycle—from retrieval to distribution, with full audit trails and anonymisation safeguards. Importantly, the system allows the secure integration of both textual and image-based records, ensuring that consent documentation and dissection photographs are digitally linked for accurate verification.

This digital transition marked a significant step forward, enhancing the traceability and integrity of BBS’s donor and tissue records and positioning BBS for future national and global collaborations by maintaining data documentation practices that conform to international biobanking standards.

## Laboratory processes/constraints

Every single brain retrieved, including the CSF, is a precious, non-renewable resource and the lab must ensure proper storage and strategically divide it to ensure that it serves various purposes. The brain is semi dissected, with one half fixed in formalin and the other flash-frozen upon slicing and cutting into blocks. The rapid freezing of the fresh brain blocks in isopentane cooled by dry ice ensures that the integrity of RNA and proteins are maintained, validated by its RNA Integrity Number (RIN) and protein quality, which is crucial for advanced molecular studies such as RNA sequencing and proteomics. An overview of the list of neuropathological findings currently held by BBS is summarised in Table 1.

**Table 1:** Overview of neuropathological findings currently held by Brain Bank Singapore.

Donor’s age	Neuropathological diagnosis
75	High-grade diffuse glioma, Intermediate AD Neuropathologic change
73	Neocortical Lewy pathology, Intermediate AD neuropathologic change
61	Neuronal loss and gliosis, Presence of microinfarcts
79	High AD neuropathologic change
64	Motoneuron disease (ALS-TDP)
67	Metastatic adenocarcinoma
71	Normal brain for age
101	Subacute haemorrhagic infarct in the right middle cerebral artery territory ARTAG.
82	High AD neuropathologic change Amygdala-predominant Lewy body related a-synuclein pathology LATE (Limbic-predominant Age-related TDP-43 Encephalopathy) Chronic right subdural haematoma.
79	Neocortical Lewy Pathology α-Synuclein oligodendroglial pathology either representing concomitant “minimal change MSA” or Lewy Body disease with prominent oligodendroglial pathology LATE (Limbic-predominant Age-related TDP-43 Encephalopathy)
60	Multiple System Atrophy (MSA)

Researchers frequently request specific neuroanatomical regions (hippocampus, substantia nigra, prefrontal cortex); therefore, consistencies in brain dissection and knowledge of morphological sites are crucial. Brain dissection must be performed in an identical fashion every time to ensure comparability across samples and training staff to this level of precision is difficult and time intensive. Our lab personnel attend a short exchange programme with the Multiple Sclerosis and Parkinson’s Tissue Bank in UK to emulate best practices on dissection, cryotomy and microtomy techniques as well as immunohistostaining procedures. To ensure that researchers get fair and equal access to these tissues, BBS maintains a Tissue Access Committee (TAC) comprising both scientists, clinicians and layman donors which will oversee tissue requests and allocate scarce resources based on a peer-reviewed application process to the most meritorious research projects.

Within the BBS’s facility, sections of anatomically important regions are prepared and stained slides are sent to our corresponding neuropathologist for a definitive clinical diagnosis. Shipping of these fixed tissues is done in specialised containers in compliance with local and overseas shipping regulations, which could be complex, expensive and risky. Efforts are underway to engage and develop local neuropathology expertise.

Safety procedures are of paramount importance and safety controls within the lab needs to be reviewed periodically. This minimises the risk levels exposed to the lab personnel, which can be hazardous and have a lasting effect on the staff.

BBS is jointly funded by six partner institutions through a Memorandum of Agreement renewed every three to 5 years. While this shared model promotes accountability and co-ownership, it also introduces financial uncertainty, as any partner may withdraw support at the end of a funding cycle. Efforts to establish a more sustainable model, including seeking national-level support and philanthropic contributions, are being explored.

### Challenges in securing national-level funding

While the BBS initiative aligns with Singapore’s broader research objectives, as a national resource the BBS primarily provides infrastructure and biospecimen support rather than direct research output, and hence BBS falls outside the scope of most currently available funding mechanisms.

### Challenges in establishing a unified philanthropic model

Attempts to secure philanthropic donations are complicated by the multi-institutional structure of BBS, which makes it challenging to establish a dedicated donation channel. While there was shared recognition of the importance of developing sustainable philanthropic support for BBS, the coordination of donation mechanisms across multiple institutional frameworks required careful alignment of governance, financial oversight, and operational processes, which proved complex to implement in practice.

Despite these constraints, BBS has begun developing fundraising pathways to sustain its operations. A public donation page hosted on NTU’s giving platform now enables the members of public to donate directly to BBS. In parallel, BBS has initiated a phased outreach strategy, that includes digital engagement with registered brain donors and placement of donation links at community outreach booths. This groundwork has supported the transition to more active fundraising efforts, including the launch of BBS’s inaugural public fundraising event scheduled for the end of 2025. These initiatives provide a feasible pathway for public donation to come in while broader multi-institutional discussions on establishing a more centralised, national funding framework continue.

## Final reflections

Over the past 6 years, BBS has evolved from a pioneering concept into a nationally recognised tissue bank, underpinned by concerted multi-institutional effort and collective vision. Its journey has been met with challenges that centre around cultural sensitivities, procedural constraints and some uncertainties regarding long-term sustainability.

Despite these challenges, BBS has established a strong governance foundation across six major healthcare and academic institutions who continue to pledge their support, maintaining a well-documented and ethically managed donor registry and an expanding tissue repository that supports pilot research studies.

In the coming decade, BBS aims to expand its donor pool and strengthen its role as a national platform for education on brain health, ageing and mental wellbeing. Public outreach and donor engagement will continue to be central pillars in building trust and understanding around post-mortem donation. A key focus will also be on recruiting donors with mental health conditions, a unique and underrepresented group in global brain banking, to help address critical gaps in neuropsychiatric research.

Looking ahead, BBS aspires not to only serve Singapore’s neuroscientific community but to also contribute to global research networks focused on Parkinson’s disease, dementia and mental health. These lessons learned over the past 6 years have strengthened and reaffirmed its purpose to advance ethically governed brain banking in Asia.

## Data Availability

The raw data supporting the conclusions of this article will be made available by the authors, without undue reservation.

## References

[1] Bell JE Alafuzoff I Al-Sarraj S Arzberger T Bogdanovic N Budka H . Management of a twenty-first century brain bank: experience in the BrainNet Europe consortium. Acta Neuropathol. (2008) 115:497–507. doi: 10.1007/s00401-008-0360-8, 18365220

[2] Liu F Yin XS Cong C Wang Y Ma C. Human brain banking as a convergence platform of neuroscience and neuropsychiatric research. Hum Brain. (2022) 1:46–62. doi: 10.37819/hb.001.001.0204

[3] Singapore Department of Statistics. Population in Brief 2024 [Internet]. Singapore Ministry of Manpower & National Population and Talent Division. (2024). (Population in Brief). Available online at: https://www.population.gov.sg/files/media-centre/publications/Population_in_Brief_2024.pdf (Accessed December 16, 2025).

[4] buddhanet.net. Buddhist Studies: Death and Dying in the Tibetan Tradition [Internet]. (2022). Available online at: https://www.buddhanet.net/e-learning/history/deathtib2/ (Accessed December 16, 2025).

[5] LifeCenter Northwest. Buddhism and organ donation [Internet]. (2025). Available online at: https://lcnw.org/buddhism-and-organ-donation/ (Accessed December 16, 2025).

[6] Singapore Department of Statistics. Census of Population 2020 – Religion [Internet]. Singapore Department of Statistics. (2021). Available online at: https://www.singstat.gov.sg/-/media/files/visualising_data/infographics/c2020/c2020-religion.ashx (Accessed December 11, 2025).

[7] Codingest. PERCEPTIONS OF ORGAN DONATION AMONG MUSLIMS IN SINGAPORE: ETHICAL AND JURISPRUDENTIAL CONSIDERATIONS [Internet]. Islamonweb English. (2025). Available online at: https://en.islamonweb.net/perceptions-of-organ-donation-among-muslims-in-singapore-ethical-and-jurisprudential-considerations (Accessed December 16, 2025).

[8] LeFebvre R Franke V. Culture matters: individualism vs. collectivism in conflict decision-making. Societies. (2013) 3:128–46. doi: 10.3390/soc3010128

[9] Parliament of Singapore. Human Organ Transplant Act (Cap. 131A) [Internet]. (1987). Available online at: https://sso.agc.gov.sg/Act/HOTA1987 (Accessed December 16, 2025).

[10] Parliament of Singapore. Medical (Therapy, Education and Research) Act (Cap. 175) [Internet]. (1972). Available online at: https://sso.agc.gov.sg/Act/MTERA1972 (Accessed December 16, 2025).

[11] SingHealth. Hepatitis B – Conditions & Treatments [Internet]. Available online at: https://www.singhealth.com.sg/symptoms-treatments/hepatitis-b (Accessed December 11, 2025).

[12] National Childhood Immunisation Programme. Childhood immunisation programme in Singapore [Internet]. Ministry of Health Singapore. (2016). Available online at: https://isomer-user-content.by.gov.sg/3/ae540999-7983-4030-895c-b4644aa840f5/chap9_01.pdf (Accessed December 16, 2025).

[13] Tissue and Cell Bank, Antwerp University Hospital, Edegem, Belgium Caremans J Department of Translational Neurosciences, University of Antwerp Faculty of Medicine and Health Sciences, Antwerp, Belgium Valgaeren H Department of Biomedical Sciences, University of Antwerp Faculty of Pharmaceutical, biomedical and veterinary medicine sciences, Antwerp, Belgium Muylle L . Virucidal activity of formaldehyde solutions used for preservation of allograft tympano-ossicular systems. B-ENT. (2021) 16:202–8. doi: 10.5152/b-ent.2021.20119

[14] amfAR TF for AR. Hepatitis B in the Asia-Pacific [Internet]. amfAR: The Foundation for AIDS Research; (2023). Available online at: https://www.amfar.org/wp-content/uploads/2023/08/TA_Hepatitis-B-Issue-Brief_080223-v1529.pdf (Accessed December 16, 2025).

